# Assessment and management of medical emergencies in eating disorders: guidance for GPs

**DOI:** 10.3399/bjgp23X732849

**Published:** 2023-04-27

**Authors:** Faraz Mughal, Simon de Lusignan, Ulrike Schmidt, Kamaldeep Bhui

**Affiliations:** School of Medicine, Keele University, Keele; Nuffield Department of Primary Care Health Sciences, University of Oxford, Oxford; Department of Psychological Medicine, King’s College London, London; Department of Psychiatry and Nuffield Department of Primary Care Health Sciences, University of Oxford, Oxford

## Background

Eating disorders are common, affect people of all ages, and can present as medical emergencies in community, primary care, or hospital settings.

In 2017, in response to the death of a 19-year-old female with anorexia nervosa, the Parliamentary and Health Service Ombudsman produced a report entitled *Ignoring the Alarms: How NHS Eating Disorder Services are Failing Patients*.^1^ In 2019, the Royal College of Psychiatrists began work to update existing eating disorder guidance (MARSIPAN and Junior MARSIPAN) alongside expert reference groups guided by the National Collaborating Centre for Mental Health; this resulted in the new *Medical Emergencies in Eating Disorders* [MEED]: *Guidance on Recognition and Management* report.^2^ This new guidance is intended for people of all ages covering all eating disorders. This practice piece consolidates the key recommendations for GPs and primary care teams.

## How Can Eating Disorders Present?

Eating disorders present challenges for GPs on recognition and diagnosis because symptoms and signs of eating disorders can have multiple aetiologies, and are often comorbid with other mental and physical health problems.^2^ Presentations may be prompted by parent/carer, partner, school, or employer concern. Symptoms can include a change in weight: either a reduction, gain, or failure to thrive; dietary restriction; binge eating; a fear of gaining weight; body image disturbance; and compensatory behaviours such as purging including self-induced vomiting and laxative or diuretic misuse. Males should be asked about excess training/exercise and use of anabolic or androgenic steroids, and it should be noted that tall males or females may be very unwell and compromised with a reasonable BMI score. In young people weight loss can be more acute than in adults. Complications of eating disorders include amenorrhea, reflux, hair loss, arrhythmia, abnormal liver function, pancytopenia, dehydration, and electrolyte disturbances, and these may present as the first problem the GP addresses in an undiagnosed eating disorder.^3^

Differential diagnoses include malabsorption (coeliac disease or inflammatory bowel disease), malignancy, leukaemia, lymphoma, infection (tuberculosis, HIV, sepsis), endocrine disorders (hyperthyroidism, diabetes, hypercortisolism), pregnancy, mood and anxiety disorders, obsessive compulsive conditions, substance misuse, and neurodevelopmental and personality disorders.

A thorough history and examination should be conducted and appropriate tests subsequently requested (see Box 1 for essential investigations). Where an eating disorder is suspected in primary care the patient should be referred for specialist assessment. Referral criteria however should not be dependent on BMI because early warning signs may be present in someone with a normal BMI.

## When is there High Impending Risk to Life?

Most medical complications in eating disorders are a result of undernutrition and/or compensatory behaviours such as purging.

The MEED guidance has produced a traffic light system for assessing impending risk to life (Table 1 in the guidance: green suggests low risk, amber indicates high concern for risk to life, and red indicates impending risk)^2^ to support emergency management. GPs and primary care clinicians should assess patients with eating disorder symptoms in person to complete a full risk assessment and consider family/carer information, as patients may be in denial about the severity of their condition. It is important to note that people with eating disorders other than anorexia nervosa can present in an emergency (for example, haematemesis or hypokalaemia) with BMI in normal range, and that some patients may appear well, which can lead to false reassurance. In Box 2, clinical factors that have been identified as ‘red’, suggesting impending risk to life, are listed for GPs and primary care clinicians to recognise and, if present, should result in urgent specialist assessment and/or admission.

## What to Do if the Patient Refuses Admission or Referral?

A patient whose life may be at impending risk because of an eating disorder and refuses admission or referral may require a Mental Health Act assessment. The GP should sensitively broach this possibility with the patient and family, and, where needed, discuss this with the emergency mental health team, and facilitate the sharing of appropriate documentation.

## Where Should Care Take Place?

If the diagnosis is clear and risk to the patient is low, the primary care team can monitor and support the patient and the family until the patient is assessed in secondary care. If the risk is moderate or high, the patient should be referred urgently to A&E or an acute psychiatric or eating disorders unit. In patients who become unwell during refeeding, GPs should consider refeeding syndrome (peripheral oedema/acute fluid overload, hypokalaemia/hypophosphataemia, or organ dysfunction: cardiorespiratory failure or deranged liver transaminases) and communicate with the treating team. From 2020/2021, 95% of children and young people should receive treatment for an eating disorder within 1 week where urgent, and 4 weeks if non-urgent in dedicated eating disorder services.^4^

## Support for Families and Carers

The families and carers of patients with an eating disorder may feel helpless, anxious, and concerned while the patient is unwell and receiving treatment. They do however usually have an important role in the person’s recovery and a collaborative approach should be encouraged. GPs should listen and understand their concerns, and signpost to educational resources such as from the charity Beat (https://www.beateatingdisorders.org.uk/).

## Where Should Medical Monitoring of Patients Occur?

Eating disorders are severe mental illnesses and the guidance recommends that physical health monitoring (such as the blood tests listed in Box 1) should occur in primary care in collaboration with medical and eating disorder services.^2^ Local policies should be agreed in integrated care systems to prevent patient safety incidents and these may include the development of shared care protocols and new funding for primary care for the monitoring of these patients.

## Type 1 Diabetes and Disordered Eating

The criteria for type 1 diabetes and disordered eating (T1DE) are type 1 diabetes, intense fear of weight gain, and inappropriate recurrent restriction of insulin causing diabetic distress and impairment of daily functioning. Red flags can include recurrent ketosis or hypoglycaemia, several hospital admissions, disengagement from diabetes care, and not requesting medications. Long-term diabetic complications are a concern, and a multidisciplinary approach is needed including diabetologists, specialist nurses, psychiatrists, and GPs.

## Box 1 Key actions for a suspected eating disorder

- Building a relationship with the patient (especially where there may be denial or a third-party consultation) to facilitate making a referral.
- Blood pressure (lying and standing), pulse.
- Temperature, weight (kg), electrocardiogram.
- Bloods: full blood count, urea and electrolytes, liver function tests, bone profile, phosphate, glucose, thyroid function tests, magnesium, creatine kinase.

## Box 2 ‘Red’ factors on risk assessment

- Rapid weight loss (obesity or ARFID) or >1 kg/week for 2 weeks.
- BMI of <13 in over-18s and m%BMI <70% in under-18s.
- Heart rate <40 b.p.m.
- Blood pressure <0.4 centile for age, <90 mmHg if >18 years, and syncope and postural drop.
- Fluid refusal, severe dehydration, or acute food refusal.
- Temperature <35.5°C tympanic.
- ECG changes = prolonged QTc interval.
- Unable to sit up from lying flat or get up from squat.
- Multiple daily episodes of vomiting and/or laxative misuse.
- Severe self-harm with high risk of death by suicide.
- Poor insight or motivation or fear leading to weight gain resistance.
- High levels of exercise (>2 h/day) when malnourished.
- Hypokalaemia (<2.5 mmol/L), hypoglycaemia (<3.0 mmol/L), hyponatraemia/hypocalcaemia/hypoalbuminaemia/hypophosphataemia.
- Anaemia (Hb <10 g/L) or liver transaminases >3 × normal range.
- Life-threatening medical condition: acute confusion, haematemesis, upper gastrointestinal perforation, diabetic ketoacidosis, significant alcohol intake.
- HbA1c >10% in patients with diabetes.

*ARFID = avoidant restrictive food intake disorder.*

## Summary

GPs often assess people who are likely to have an eating disorder and management can be challenging. Medical emergencies in people with eating disorders can be fatal. It is important that GPs are aware of the clinical factors that suggest impending risk to life and, if identified, organise urgent specialist assessment and/or admission.
